# Procedural Requirements and Recommendations for Multiplex Immunofluorescence Tyramide Signal Amplification Assays to Support Translational Oncology Studies

**DOI:** 10.3390/cancers12020255

**Published:** 2020-01-21

**Authors:** Edwin Roger Parra, Mei Jiang, Luisa Solis, Barbara Mino, Caddie Laberiano, Sharia Hernandez, Swati Gite, Anuj Verma, Michael Tetzlaff, Cara Haymaker, Auriole Tamegnon, Jaime Rodriguez-Canales, Clifford Hoyd, Chantale Bernachez, Ignacio Wistuba

**Affiliations:** 1 Department of Translational Molecular Pathology, the University of Texas MD Anderson Cancer Center, Houston, TX 77030, USA; MJiang1@mdanderson.org (M.J.); lmsolis@mdanderson.org (L.S.); barbara_mino@yahoo.com (B.M.); CDLaberiano@mdanderson.org (C.L.); SDHernandez@mdanderson.org (S.H.); SGite@mdanderson.org (S.G.); AVerma2@mdanderson.org (A.V.); CHaymaker@mdanderson.org (C.H.); ATamegnon@mdanderson.org (A.T.); cbernatchez@mdanderson.org (C.B.); iiwistuba@mdanderson.org (I.W.); 2 Department of Pathology, Division of Pathology and Laboratory Medicine. The University of Texas MD Anderson Cancer Center, Houston, TX 77030, USA; MTetzlaff@mdanderson.org; 3 Translational Medicine, Oncology Research & Development, AstraZeneca, Gaithersburg, MD 20878, USA; jaime.rodriguezcanales@astrazeneca.com; 4 Akoya Biosciences, Hopkinton, MA 01748, USA; choyt@akoyabio.com; 5 Department of Melanoma Oncology, the University of Texas MD Anderson Cancer Center, Houston, TX 77030, USA; 6 Thoracic/Head and Neck Medical Oncology, the University of Texas MD Anderson Cancer Center, Houston, TX 77030, USA

**Keywords:** tumor immune profiling, multiplex immunofluorescence, tyramide signal amplification, image analysis

## Abstract

In the development of a multiplex immunofluorescence (IF) platform and the optimization and validation of new multiplex IF panels using a tyramide signal amplification system, several technical requirements are important for high-quality staining, analysis, and results. The aim of this review is to discuss the basic requirements for performing multiplex IF tyramide signal amplification (TSA) in formalin-fixed, paraffin-embedded cancer tissues to support translational oncology research. Our laboratory has stained approximately 4000 formalin-fixed, paraffin-embedded tumor samples using the multiplex IF TSA system for immune profiling of several labeled biomarkers in a single slide to elucidate cancer biology at a protein level and identify therapeutic targets and biomarkers. By analyzing several proteins in thousands of cells on a single slide, this technique provides a systems-level view of various processes in various tumor tissues. Although this technology shows high flexibility in cancer studies, it presents several challenges when applied to study different histology cancers. Our experience shows that adequate antibody validation, staining optimization, analysis strategies, and data generation are important steps for generating quality results. Tissue management, fixation procedures, storage, and cutting can also affect the results of the assay and must be standardized. Overall, this method is reliable for supporting translational research given a precise, step-by-step approach.

## 1. Introduction

In recent years, emerging evidence has revealed the importance of the relationship between the response to cancer immunotherapy of immune cells in the cancer microenvironment [1,2,3,4,5,6]. This immune response against cancer at the cellular level has a clear role in fighting cancer progression and producing immune resistance. The complexity of studying these cancer tissues demands new systems to allow the analysis of cell phenotypes in their microenvironment as well as their spatial distribution [7]. Although various sophisticated multiplex immunohistochemistry (IHC) methods are available for analyzing formalin-fixed, paraffin-embedded (FFPE) material, the current applications have limited scalability and throughput, because even with high levels of multiplexing, the analysis is limited to small regions of interests and/or a limited number of fields of view [8] The immune profiling analysis of tissue samples from FFPE biopsies has become a key tool for understanding the complexity of tumor immunology and discovering novel predictive biomarkers for cancer immunotherapy. Immune profiling analysis of tissues requires the evaluation of combined markers, including inflammatory cell subpopulations and immune checkpoints, in the tumor microenvironment [9].

Multiplex immunofluorescence (IF) methods to simultaneously detect different types of molecules have been revolutionizing IHC in recent years [10], as they can identify multiple biological markers on a single tissue sample [7]. With this technique, individual cells can be assessed with extraordinary fidelity according to the antibodies included in the panel, almost equal to the fidelity of assessment seen in the bulk population, such that even rare cell populations can be studied. This technology therefore has an important role in translational oncology research [11,12,13,14,15]. Thus, multiplexed IF can help characterize the topography of immune cells in cancer in detail, including the relative localizations and interactions of marker expression on cancer cells, immune cells, stromal cells, and endothelial cells, furthering our understanding of the disease [8,16]. This approach can reproducibly quantify multiple protein levels and functional activities in intact tissue specimens. It might also be applicable to not only cancer but also other diseases, and it is well suited for prognostication at early stages of pathogenesis, when key signaling protein levels and activities are perturbed [17].

The most important sources of samples for ongoing patient care and for translational studies using these technologies are still, in most cases, archival paraffin blocks of tissue collections and, in recent years, small biopsies such as core needle biopsies used in longitudinal studies. Because the available samples often have been collected using a variety of methods, multiplex IF technique harmonization is essential to obtain staining varies minimally between different research efforts and is high-quality, to avoid misinterpretation of the results. In recent years, multiplex IF using a tyramide signal amplification (TSA) platform arose as a reliable assay capable of assessing several labeled biomarkers in one sample [18].

Multiplex IF platforms have arisen to detect multiple biological markers on a single tissue sample or an ensemble of different tissue samples [7]. Multiplex IF is important to translational oncology research because this technique can easily assess individual cells according to the antibodies included in the panel and can be used to discover rare cells. Among the most popular multiplex IF techniques is the TSA method, described by Bobrow and colleagues in the 1990s [19,20]. The method is an enzyme-linked signal amplification that detects and amplifies low copy numbers of proteins present in tissue using a conventional IHC protocol. In recent years, Akoya/PerkinElmer developed the Opal workflow, which allows simultaneous staining of multiple biomarkers within a single paraffin tissue section using TSA methodology that allows the use of antibodies raised in the same species (Supplementary Figure S1). Different panels (seven or nine markers per panel) can be created and combined to target specific biomarkers using this technology [14,21], providing a comprehensive system for cancer studies. However, rigorous steps are needed to obtain reproducible, reliable, and high-quality staining data.

**Figure 1:**
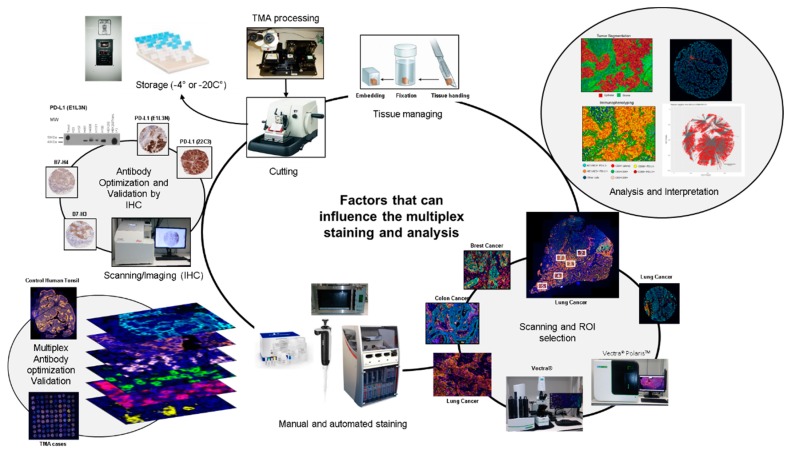
Factors that can influence multiplex image staining and analysis. Digital image analysis platforms can be influenced by factors related to tissue processing and handling, including cutting and storage. Antibody optimization and validation by immunohistochemistry (IHC), immunofluorescence (IF), and multiplex IF as well as staining, scanning and analysis procedures must be standardized to obtain accurate data.

Our Immunoprofiling Laboratory at The University of Texas MD Anderson Cancer Center is currently working with this technique and has had the opportunity, during 2015 through 2018, to stain and analyze 4142 samples, using different panels for immune profiling of paraffin tissues. We have analyzed over 20,000 regions of interest (ROIs), as well as staining slides for panel design and optimization and validation of antibodies by single chromogenic IHC, single IF, and multiplex IF (Supplementary Table S1). On the basis of our experience, this review will describe the minimal conditions for biomarker validation, tissue staining preparation, staining, scanning, ROI selection, and analysis to obtain consistent results using the multiplex IF TSA platform (Figure 1). Common problems that we observed in these different steps will be discussed, and recommendations will be proposed to improve the quality of each step, increasing the potential use of this assay for future applications in cancer immunotherapy, biomarker discovery, and precision medicine.

## 2. New Panel Design of the Study

There are several technical requirements for developing an automated multiplex IF imaging platform: (1) the ability to quantitate multiple markers in a defined region of interest, (2) rigorous tissue quality controls, (3) a balanced multiplex assay staining format, and (4) experimental reproducibility [17]. After a platform is developed, creating a new multiplex IF panel for a project requires the selection of appropriate antibodies. These antibodies should be chosen by a multidisciplinary team, including pathologists, oncologists, and immunologists, to ensure that the panel will appropriately address the aims of the project. The new panel needs to be comprehensive and coherent in the cell phenotypes that will be characterized. The identification of specific cell phenotypes—for example, by combined marker expression—needs to be defined before antibody validation.

Selecting an antibody for multiplex IF requires an understanding of the protein of interest. The relevant literature should be thoroughly reviewed [22] using various sources, such as the websites Biocompare, Antibodypedia, SelectScience, UniProt, and the National Center for Biotechnology information to understand the roles of the proteins targeted by the antibodies under consideration. Some antibodies, such as those targeting HER2, estrogen receptor, and progesterone receptor, may be selected because of their clinical implications, while other antibodies, such as those targeting immune checkpoint markers [23], may be selected to answer specific scientific or research questions. Antibodies vary in their known validity and reliability: some antibodies are well known in the literature with recognized high quality performance; some are well known for their use in alternative species or unverified tissues; and some are unknown, with inconsistent or no literature evidence [24,25].

An antibody’s clonality must also be considered. Polyclonal antibodies bind to different epitopes on the same protein and are obtained from experimental animals through repetitive stimulation of the antigen. These antibodies can enhance the IHC signal, when compared to monoclonal antibodies, but may have higher background and lot-to-lot variability. On the other hand, monoclonal antibodies bind only to a single epitope and are obtained through hybridoma technology. These antibodies have more specificity and lower background and lot-to-lot variability. Recombinant antibodies, produced by recombinant DNA technology, should also be considered. Each of these types of antibodies has advantages and disadvantages that need to be considered during the optimization and validation of a new multiplex IF panel (Table 1).

## 3. Tissue Control Selection

Selection of positive and negative tissue controls for antibody optimization, as well as cell line pellets and controls with different levels of expression [23,26,27], are essential for specific antibody validation. For research antibodies that are not yet well understood, a Western blot [23] assay in positive and negative cell lines [28] can help corroborate the specificity of the antibody. Although sometimes antibodies that are suitable for FFPE tissues are not suitable for Western blot analysis and vice versa, having Western blot results can provide more confidence in the specific of the antibodies, as seen with Programed Death Ligand 1 (PD-L1) optimization and validation (Supplementary Figure S2). The cellular location and pattern of the expression (e.g., nuclear, dot-like, diffuse cytoplasmic, or complete or partial membrane staining) [29] should be identified during the optimization process to confirm that the antibody is working perfectly, and this information must be documented for unknown antibodies. Online resources such as The Human Protein Atlas can be used for comparison of optimization results.

## 4. Antibody Optimization and Validation

The optimization and validation of antibodies for multiplex IF panels can begin with optimization by chromogenic IHC, for example with the different clones of PD-L1 marker (Supplementary Figure S2). Alternatively, depending on the experimenter’s confidence and experience with the IF protocols and targets of interest, antibody optimization and validation using only IF is acceptable [30]. For antibody optimization, several steps need to be considered, including clone selection, and cellular expression and pattern of expression, to achieve higher specificity with minimal or no background along with reproducible and reliable results for pre-analytical, analytical, and post-analytical validation [28,31].

### 4.1. Uniplex IF Antibody Optimization

**Figure 2:**
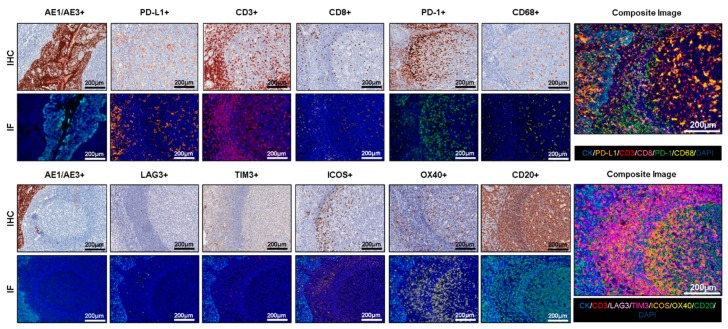
Antibody optimization and validation. Representative examples of antibody optimization and validation using conventional chromogenic IHC and multiplex IF showing similar patterns of expression by the antibodies tested in IHC and multiplex IF. Composite images show the integration of markers on a single slide. At 20× magnification.

After the necessary optimization of each individual antibody, including dilution, incubation times, and blocking, to determine the optimal conditions using IHC, antibody optimization should also be performed using IF and the TSA system to create individual protocols that will provide equivalent results between chromogenic IHC and IF in the same positive and negative tissue controls, disclosing any discrepancy or background staining [23]. The antibody dilution in most cases will be the same in IF as in IHC, but the fluorophore for each antibody needs to be carefully studied and chosen to start the process of antibody optimization by IF. In general, antibodies with relatively strong expression or with high dilution in IHC with low fluorophore intensity represent an easy starting point in this step. Each antibody needs to be tested with a fluorophore. According to our experience, the starting incubation time for any antibody is 30 min, and the starting dilution of the fluorophore conjugated to the antibody can be 1:100. These two parameters need to be tested separately to obtain similar dynamic ranges between the antibodies and their fluorophores. The ideal dynamic range of antibody fluorescence capture expression is between 50 nm and 150 nm using the Vectra/Polaris scanning system (Akoya/PerkinElmer), which means approximately 10 to 30 counts of fluorescence expression of each antibody using the inForm software (Akoya/PerkinElmer). The variation of this dynamic range needs to be optimized for each antibody conjugated with its fluorophore to ensure similar thresholds of expression for all selected markers. The use of tissue controls, such as reactive human tonsils, that reflect an exact cell subpopulation distribution is important, and we highly recommend using a set of cases from the desired primary tumor to set up the final thresholds of the antibodies and the dynamic ranges of their fluorophore expression (Figure 2).

### 4.2. Multiplex IF Optimization

The last and most crucial step for a new multiplex IF panel is to combine all the antibodies previously tested in a new multiplex protocol. Such a multiplex protocol essentially consolidates the uniplex IF protocols in a unique protocol that allows all the antibodies to be used in a specific order to obtain similar patterns to those observed in the uniplex IF staining (Figure 2). However, as there is no specific order for the different antibodies, since the order of the antibodies must be determined by the experimenter, we recommend starting the process with the antibodies that have the lowest concentrations or strongest expression, as observed in the uniplex IF protocols, and ending with the antibodies that have the highest concentrations or the lowest expression to avoid the umbrella effect. A checklist of all the steps and modifications made during the different multiplex staining tests will help track or identify possible errors during the different multiplex staining tests. As described for the uniplex IF protocols, similar dynamic ranges of each antibody’s expression should be maintained to balance all the markers in the panels with the same intensity of expression. During the multiplex staining, positive controls should be included as described above. To exclude endogenous and exogenous autofluorescence during the image analysis, the same type of tissues can be stained in parallel: with all the antibodies but no fluorophores, all the fluorophores but no antibodies, or without antibodies or fluorophores. Antigen retrieval also requires optimization and endogenous horseradish peroxidase quenching, all while ensuring complete antibody stripping and tissue integrity. In addition, properly balanced horseradish peroxidase is required to prevent TSA dimer formation, typically through titration of primary antibodies, although the dimer formation can also be reduced through titration of the secondary antibody [21]. Overall, when all these steps are followed carefully, this method is reliable and yields results that can be compared with those of uniplex IF or individual IHC antibody staining as well as other multiplex methodologies [32].

### 4.3. Staining Interferences

Cross-talking reactions between fluorophores is the most common interference that we have observed during the optimization of a panel. This interference can occur between fluorophores with similar wavelengths when one signal appears in the channel for a neighboring signal, leading to false-positives. Corrective actions include changing the position of the antibodies that produce the interference and increasing the time of the antigen retrieval related to the antibody causing the interference.

Another type of staining artifact occurs because TSA reagents covalently bind to sites surrounding the antigen, and the reagents can inhibit the binding of a subsequent primary antibody through steric hindrance, a situation known as blocking or an umbrella effect. In general, this situation occurs when multiple markers reside in a single cell compartment, such as CD3+, CD8+, and CD4+ lymphocyte membrane markers, when the tyramide from a preceding marker is deposited in the same compartment and blocks the following antigen. This phenomenon is easily detected during the optimization process through comparison with chromogenic IHC or uniplex IF using the same markers and can be corrected by increasing the primary antibody concentrations, reducing TSA fluorophore concentrations, and/or changing the order of targets in the panel. A useful approach when a staining artifact persists is to determine which antibody or fluorophore is causing the interference or blocking using the drop-control method described by Surace and colleagues [9].

## 5. Spectral Library

In parallel to antibody optimization, creation of a spectral library is necessary to detect the correct spectra excitation from each fluorophore in the multiplex IF-stained slide and avoid overlap between fluorophore excitation ranges during the analysis. Every pixel should be classified as a linear combination of spectra using a library as a reference of intensity spectra known to constitute an image (Supplementary Figure S3). In this way, the percentage of each intensity basis spectrum contributing to each pixel can be determined [33]. To generate an appropriate spectral library, uniplex IF staining needs to be performed with each fluorophore used in the panels with a primary antibody (e.g., a cytokeratin, vimentin, CD3, or CD20) without 4′,6-diamidino-2-phenylindole (DAPI) and with similar dynamic ranges, as described for the uniplex IF staining.

## 6. Pre-Analytical Interference

To avoid pre-analytical variability, the laboratory must standardize variables related to handling of the tissue and use a standard operational procedure for collection of samples and cutting. Between tissue removal and the initiation of fixation, tissue samples undergo ischemia time, meaning time without oxygen supply. During this period, the tissue suffers degradation of proteins, RNA, and DNA; activation of enzymes; and autolysis [34]. Therefore, variations in ischemia time can crucially affect IHC and multiplex IF staining (Supplementary Figure S4). Numerous reports have shown alterations in the results of estrogen receptor, progesterone receptor, HER2, procaspase, active caspase, and Ki-67 IHC due to variable ischemic times [35,36,37]. Although the American Society of Clinical Oncology and College of American Pathologists have developed guidelines for handling of tissues for estrogen receptor, progesterone receptor, and HER2 detection in breast cancer patients [38], such guidelines are not available for handling of other surgical specimens or biomarkers.

Other tissue handling practices that can make a difference in the quality of multiplex IF staining include fixing tissue as quickly as possible after resection (within less than 20 min is prudent), recommended overall sample dimensions (a suitable maximum for good fixation is 1.5 × 1.5 × 0.4 cm), an adequate volume of fixative (10–20 times the volume of the tissues for immersion fixation), and adequate time of fixation (6–18 h for small biopsy specimens as core needle biopsies and 24 h for standard samples as whole section samples approximately 1 × 1 cm.). The fixation process is another critical step in the pre-analytical validation that can interfere in the staining results. The most popular fixative used in histopathology laboratories is 10% neutral-buffered formalin, comprising 4% paraformaldehyde solution buffered to a neutral pH, in part because of its low cost and easy preparation. However, the formalin fixation process can be influenced by temperature, time, penetration rate, specimen dimension, volume ratio, pH of the buffer, and osmolality, it is a controllable step. Formalin fixation creates cross-links with peptides in the tissue by formation of hydroxymethyl groups on reactive amino acid side chains and preserves tissue morphologic characteristics with very few alterations. In addition, the duration of the formalin process can mask or damage some antibody-binding sites, decreasing antigenicity, but some antigen retrieval methods or amplification systems can help the link the antibodies with their epitopes [34,35]. Although there is a lack of available guidelines to establish a standard practice across pathology laboratories in general, we recommend following the basic rules suggested above to obtain good staining results.

Biomarker tests that will be used in tissues that undergo additional procedures before the fixation process, such as decalcification, present additional challenges, such as the need for more additional tests of the antibody of interest in this type of tissues to obtain similar results as tissues without decalcification procedures. In this situation, the panel needs to be re-optimized using decalcified tissue [31]. However, it is a challenge to find decalcified tissue with the right positive control, especially if the antibody is not expressed at higher frequencies or higher levels.

Finally, on the basis of our experience, we recommend a tissue section thickness of 3 to 4 μm for multiplex IF staining to minimize the geographic cellular distribution of the markers and to avoid cell overlap during the analysis in the same sample. An experienced technician is needed to avoid rips and folds of the tissue and to maintain similar tissue orientation across sequential slides. Although we can control the cutting procedure, most of the time we cannot control the collecting and storage conditions, so quality control of the pathology samples that are processed in multiplex IF needs to be established to avoid poor-quality staining and results.

## 7. Tissue Quality Components

We have noticed that in translational research, there is more demand for performance of this type of multiplex IF staining in small biopsies than in whole sections and we are frequently challenged to perform immune profiling on relatively scant samples. However, larger tumor samples do not present many problems with staining or evaluation because they have abundant material to choose from for the analysis. Still, we have observed that certain components or characteristics of the samples can affected the multiplex IF staining, especially in small biopsies. In our experience, surgical resections containing abundant fat tissue, as observed in breast cancer samples, can be difficult to stain. In general, an abundant fat component contributes to detachment of the material during the staining procedure, probably because the tissue does not have a good support component. Abundant necrotic areas are always a challenge for staining and analysis procedures, especially when they are between tumor. In cases with large necrotic areas, as observed in tumors treated with neoadjuvant therapies, the best option is to exclude those areas as much as possible during the analysis, although the quantity of the material analyzed may then needs to be evaluated as a limitation of the study. Bone marrow samples processed using decalcification methods can also be problematic during the staining. Decalcification procedures can alter the cells and the staining pattern, as mentioned above.

Good staining is more challenging with small biopsies, such as core needle biopsies available in longitudinal studies. In these biopsies, the presence of fat and necrosis, as well as cartilage or bone, which can affect the success of the staining depending on their proportions, are more problematic because of the small amount of the samples (Table 2). Material secreted by tumors, such as mucus, can interfere with the quality of the staining, limiting the quality of the analysis and the results. The quantity of the tumor content can also interfere in the analysis, especially in core needle biopsies. However, there is no specific threshold of tumor content in small biopsies to determine whether the tissue is adequate for multiplex IF immune profiling. We recommend a threshold greater than 100 malignant cells to minimize the risk of errors in the analysis and interpretation of the samples. In addition, it is important that the tumor compartment in small biopsies be at least 10% of the entire biopsy in a minimum sample size of 10 mm × 2 mm to qualify for multiplex IF staining.

The multifragmentation of small samples should also be considered. In this situation, these cases must be considered individually, and we must determine whether we will be able to obtain enough representative, quality staining of the material. In our experience, if the fragments of the sample are approximately 2 mm × 2 mm each or less, success of the staining will be very limited, compromising the quality of the results. All these characteristics can be considered as criteria for exclusion for staining and analysis (Table 2).

## 8. Pathology Quality Control for Selection of Oncology Samples

**Figure 3:**
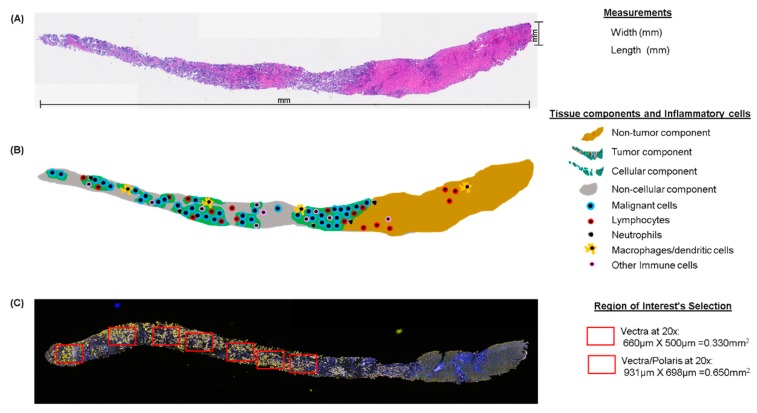
Microphotographs of hematoxylin and eosin (H&E), multiplex IF, and schematic representation of tissue quality components in longitudinal studies. (**A**) Principal measurements on a core needle biopsy with H&E. (**B**) Schematic of the same core needle biopsy showing the principal compartments and inflammatory evaluation during the pathology quality control of the sample. (**C**) Multiplex IF microphotograph showing the selection of regions of interest in the same core needle biopsy. H&E and multiplex IF are at 4× magnification.

To minimize these pre-analytical variables, a pathology quality assessment is a necessary initial step. Quality assessment of biopsy samples by a well-trained pathologist to determine various histologic parameters including the dimensions of the sample and the locations of the different cell types comprising the sample (Figure 3) helps ensure high-quality results from multiplex IF and guides the use of resources by identifying high-quality samples for multiplex IF. For oncologic samples, the pathologist must first confirm the diagnosis; in cases with a discordant diagnosis revealed by the pathologist, a second opinion is needed from a specialist in the tumor type observed to avoid errors in the analysis, especially for rare tumors. There are currently no guidelines for assessing the quality of tumor samples for multiplex IF staining. In our experience, sample size, tumor content, and other characteristics can predict the quality of the staining and analysis. We identify these characteristics as follows: (1) Using a simple hematoxylin and eosin stain, the tissue is measured for length and width (Figure 3A), then divided into tumor and non-tumor compartments. (2) The tumor compartment is then divided into a cellular component and a non-cellular component, including necrosis, mucin, and fibrin. (3) The cellular component of the tumor compartment is further sub-divided into malignant cells and non-malignant cells, including inflammatory cells. (4) The number of tissue fragments is recorded so that representative areas from the entire tumor compartment can be analyzed. (5) The percentage of inflammatory cells among the non-malignant cells in the tumor compartment, not including any ulcerated tumor areas, is scored as low (<25%), medium (25–75%), or high (>75%) infiltration to provide a preliminary characterization of the tumor microenvironment (Figure 3B). This pathology quality assessment thus helps triage out cases with inadequate malignant cells or abundant cellular and non-cellular components that could reduce the quality of multiplex IF staining and the interpretation of the results.

## 9. Area Selection for Multiplex IF Analysis

**Figure 4:**
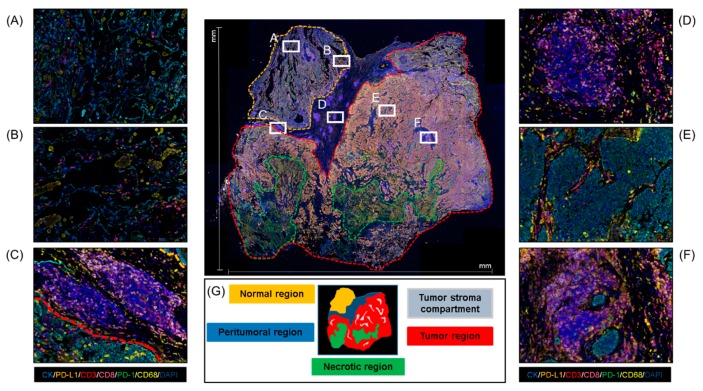
Whole-section sample microphotograph of multiplex IF and the different areas that can be analyzed in this type of material. (**A,B**) Normal regions. (**C**) Peritumoral region. (**D**) Tertiary lymphoid structures. (**E**) Tumor region. (**F**) Aggregate lymphoid region. (**G**) Schematic of regions on a whole-section sample. At 4× magnification of the panoramic view and 20× magnification of the specific regions.

In the field of multiplex IF, the use of scanners represents a major technological advance that enables the use of multiple sometimes unstable fluorochromes and thus more than seven different antibodies on the same slide [16]. A Vectra or Vectra Polaris scanner allows extremely precise imaging in brightfield or fluorescence detection with high resolution by combining multiband filter cubes, which increase the flexibility of the multispectral camera [39]. Using these scanners, we can analyze whole-slide images or specific ROIs. Confining the image analysis region to one or more smaller ROIs is often necessary to create an accurate and computationally viable method for tissue image analysis (Figure 4) [40]. Because the strategy of the analysis must be specified according to the aims of the study, the ROIs must be selected at the beginning of the project. The ROI selection methodology, including whether the analysis was based on an ROI, hot spots, whole-slide images, or a pre-selected sample, should be described transparently to be reliable and reproducible. Different ROI selection approaches are subject to different potential errors, which can impact the study design [41]. However, there is no established guidance on the quantity of tissue from the sample that can be considered representative for image analysis. In our experience, ROI selection is more challenging in smaller samples, such as core needle biopsies, because the small area represented by the biopsy limits the possible ROIs that can be chosen for the analysis. Overall, we recommend selecting the entire tumor area (Figure 3C).

Detection and phenotypic characterization of cells in ROIs require both the scanner system and image analysis software, such as the inForm software (Akoya/PerkinElmer). Image analysis software allows automatic analysis of parameters that cannot be accurately discerned by the human eye (e.g., cell forms, multiple cell phenotype networks, and vascular network) [16]. InForm in particular includes a user-trainable algorithm for tissue analysis based on morphology as well as specified markers. Training is usually performed using quick iterations and adjusted until optimal results are obtained. To begin the analysis of a tissue image using the trainable algorithm when there are multiple ROIs, the tissue can be divided into compartments using the tissue segmentation tool, by which a training area can be drawn for each category, e.g., tumor, stroma, or glass. Tumor markers (e.g., cytokeratins, S100, or glial fibrillary acidic protein) are used to differentiate the tumor compartment from the stroma compartment when possible. We can refine the segmentation by adding new training areas to address any miscategorized tissue (Supplementary Figure S5). The algorithm then analyzes all tissues included in the inclusion annotation of the ROIs. Areas of disinterest, such as necrosis, tissue artifacts, and staining artifacts, must be excluded with the disinterest tool [40].

After tissue segmentation, cell segmentation can be performed using DAPI as counterstaining to phenotype individual cells. For cell segmentation, the algorithm allows us to combine the expression of different markers according their compartment of expression, such as nucleus, cytoplasm, or membrane, to more accurately identify each cell. However, we always recommend including a universal membrane marker (e.g., a cocktail of membrane markers) in the panel to better characterize the cell segmentation and to optimize the splitting sensitivity and cytoplasmic thickness to achieve perfect cell segmentation (Supplementary Figure S6).

After cell segmentation, we are ready to add and train the algorithm for cell marker identification using a phenotyping tool. In general, our approach is working with individual marker expression, or analyzing mutually exclusive markers (e.g., cytokeratin, CD3, and CD68) in a first run, and then adding a new marker in each consecutive run of analysis and training sessions to create an individual algorithm for the different markers (Supplementary Figure S7). However, for cellular marker identification, the software requires only five examples per marker. For tissue immune profiling across multiple samples, we have found that at least 30 examples of each cellular marker are necessary for reliable specific cell identification. This number of examples may involve an initial training with 5-10 examples for each cell marker followed by the addition of more examples of the specific cell that express the marker to improve cell iteration identification and confidentiality to avoid the cell background and false positivity. When the training for each marker is complete and saved, we can apply the algorithm of each marker in a set of cases to carefully verify that the algorithms are working properly in our samples. The education of the software may be time-consuming, with a phase of learning or “teaching” [16]. Using this approach, the optical signal from each biomarker in the spectral unmixing image can be isolated, assessed separately, and quantified [42] to integrate the data at the end of the process, using R studio software with the phenoptr program (Akoya/PerkinElmer). Finally, although the software allows automatic batch analysis using the created algorithms in many ROIs at a time, we generally do not recommend this approach because of the high variability that we see in marker expression and sample collection (differences in tumor histology, tumor subtypes, and the need to remove areas of disinterest from each ROI). For quantitative image analysis, the software needs to be accessible, with easy automated detection capabilities, including tissue segmentation, compartmentalization of staining (nuclear, membranous, or cytoplasmic), and spatial localization of cell distribution, which are critical for studying the different markers included in different panels [39].

## 10. Data Report

The main reason for performing a multiplexed assay is to obtain a tabulated report format with a high volume of tumor biological information representing multidimensional data on tissue architecture, co-expression of markers, spatial distribution of multiple cell phenotypes, and identification of rare cell types [38,43]. For multiplex IF analysis, semiquantitative scoring is usually not appropriate because of the massive amount of information in a single slide and because the power of multiplex IF platforms is reflected in the identification of densities of cell phenotypes that can be associated with pathology, clinical patient information, and prognoses [44,45]. Automated computational pathology platforms are a promising direction for more objective quantitation of pathology staining [46]. Quantitative cell density assessment from tissue of multiplexed staining using this technology has the potential to produce data that are more rigorous and on a continuous scale, allowing for more precise correlations to clinical or biological data. In quantitative data reporting, the signals on the slide are assumed to be representative and quantitatively related to the amounts of the different antigens in the sampled section of tissue, which is in turn related to the absolute amount of the antigen in the tissue as a whole [21]. The data report can be presented as a ratio, such as the amounts of antigen expression or cell densities relative to the area in which the targets of interest are expressed, by millimeter squared [21,47]. Another aspect of the data is spatial analysis (Supplementary Figure S8) based on information such as location, distance, direction, or topology, as well as classification and neighborhood analysis and clustering, which assembles objects with similar characteristics and simplifies and enriches the structure of the data [39,48]. Essentially, a spatial analysis uncovers underlying patterns in the cell data analysis and can be built on in future research. The images generated by the multiplex IF generally need to be delivered as a part of the data report to illustrate and to accurately identify the different cell phenotypes in the different samples (Supplementary Figure S9).

## 11. Quality Control Assessment and Analytical Validation

The digital pathology image analysis tools applied using software can provide quantitative results in an automated, high-throughput manner once the trainable algorithm has been optimized for the application of interest [32]. However, this technique requires proper high quality control of the data. A key first step for data quality control is the verification of the multiplex stain (both patient tissues and external positive and negative control tissues) by a pathologist to determine its acceptability or the need to repeat the stain [21]. During the image analysis process, the algorithm modified for quantification purposes needs to be carefully verified to ensure that the training of the algorithm is sufficient to accurately capture the total numbers of cells in the different ROIs during the image analysis (Supplementary Figures S5–S7). In addition, it is necessary to test whether the algorithm can identify and differentiate cell expression with an error no greater than 5%. The data need to be reviewed by a pathologist familiar with the methodology and made consistent and reproducible across the samples using the trainable image analysis software. Adequate internal positive controls are the best way to exclude false-negatives, and good internal negative controls are the best way to rule out false-positives. If the staining of control tissue is low quality, the multiplex IF slides stained in that run are excluded from analysis. Analytical validation of the different panels also consolidates the reproducible information generated by the laboratory. Sequential slides should be stained consecutively for at least two time points and at least 10 cases to ensure reproducibility and minimal variation to optimize the quantification performance. Data that have undergone these procedures provide a high-quality main core to build the scientific, technical, and medical knowledge to improve future understanding.

## 12. Data Interpretation

Final data interpretation should again involve the expertise of a pathologist but can be performed by a larger team with specialty expertise in various areas relevant to the objectives of the analysis [40]. Owing to the large amounts of data generated, we advise including a statistician, biostatistician, or computational mathematician on the team. Once tissue has been analyzed using the selected ROIs, different levels of information can be retrieved. The expression of different biomarkers of interest can be accessed at the level of individual cells providing information about which cell types and biological processes are present on the sample. Furthermore, spatial distributions of different cell populations can be analyzed to relate the biological activities of these different phenotypes to the morphologic context of the samples. The continuous variables in the quantitative analysis generated by the multiplex IF assay can be integrated with clinicopathologic variables to stratify patients according to the multiplex IF results. Some commercially available algorithm solutions come with data interrogation and plotting tools to aid in this stratification better data interpretation. Similarly, some image analysis service providers offer further statistical analysis and expert interpretation of study data as part of the service package [40]. However, there is no standardized method for setting the cut-off values, although the median expression level of each biomarker is a commonly selected cut-off, and sometimes tertiles or quartiles can be analyzed. This lack of standardization sometimes causes inconsistent results between similar studies. Pathologists should be cautious when comparing different assay data between studies and need to standardize the data when possible to avoid inter-observer variation [49,50]. Furthermore, ensuring inter-site reproducibility [51] is important because the multiplex IF technique has potential use in clinical scenarios.

## 13. Conclusions

Multiplex IF TSA platforms have become indispensable tools for pathologists from basic to translational research for elucidating the pathophysiology of cancer. This type of platform can be a powerful tool for discovery of biomarkers that eventually lead to personalized medicine. Even though the multiplex IF procedure has recently been automated, there remain many considerations for optimizing multiplex IF TSA effectively and interpreting the results appropriately. Optimization of multiplex IF not only in tissue sections as well as in cell blocks from liquid biopsies, as recently described by Roy and colleagues [52], is particularly important for newly discovered molecules or pathways in oncology. The specificity and sensitivity of this technique need to be validated, as does the consistency of the method to stabilize inter-observer variation and produce more objective interpretation of the results. The interpretation of multiplex IF data needs to be carefully planned and to involve the participation of a well-trained multidisciplinary team, including technicians, pathologists, oncologists, immunologists, informaticians, and biostatisticians.

## Figures and Tables

**Table 1:** Antibody properties and comparisons.

Properties	Monoclonal Antibody	Polyclonal Antibody	Recombinant Antibody
**Epitope selectivity**	Generated by a single B-cell line and thus recognize only a single epitope of a protein of interest	Mixture of antibodies that all recognize different epitopes of the protein of interest	Antibodies created to recognize a specific epitope of a protein of interest
**Source**	Mouse or rabbit	Variety of species including mouse, rabbit, goat, sheep, and donkey	Entirely animal-free production process
**Reproducibility**	More reproducible generated immortal B-cell hybridomas	Prone to batch-to-batch variability (produced from animal sera)	High reproducibility and guaranteed continuity of availability without any dependence on animal immunization
**Cross-reactivity**	Less likely to cross-react with other proteins and lower background staining	May contain non-specific antibodies and background staining	No background staining
**Specificity/sensitivity**	Highly specific owing to single target epitope but less sensitive because often unable to detect masked antigen	More sensitive owing to targeting of multiple epitopes of an antigen but less specific than monoclonal antibodies	Highly specific and sensitive
**Challenges**	More challenging to work with when looking at low-abundance proteins or proteins with high variability	Poor choice for long-running studies	Last resort owing to higher cost

**Table 2:** Recommended baseline sample exclusion criteria for longitudinal studies.

Tissue Characteristic	Hematoxylin and Eosin
Size	Less than 2 × 2 mm
Fragmentation	Multi-fragmentation
Tumor content	Non-malignant cells or fewer than 100 malignant cells in the sample*
Fibrosis	Fibrotic tissue without inflammatory cells
Necrosis	Necrotic tissue or malignant cells surrounding with necrosis with any parenchymal sustentation
Previous procedures	Decalcification procedures that can alter the quality of the staining**
Preservation	Staining artifact of oxidation/desiccation
Cellular characteristics	Crushed cells artifact
	**Multiplex immunofluorescence**
Size (vectra) ^‡^	Minimum total area of five regions of interest (each 660 × 500 µm, at 20×) or 1.65 mm^2^ of total area analyzed***
Tumor content	Non-malignant cells or fewer than 100 malignant cells in the total area of analysis
Inflammation	Non-inflammatory cells or fewer than 10 cells expressing the principal marker in the entire area analyzed (e.g., CD3)
Fibrosis/necrosis	Exclusion’s criteria when interfere in the analysis.
Tissue/cellular characteristics	Several folds, crushed cells, overlapping, or mucinous tumoral secretion ^†^

* Presence of malignant cells are not necessary in the post-treatment biopsies. ** These cases need specific marker validation in the panels that are not affected for the decalcification procedures. ^‡^ The region of interest of a Vectra Polaris scanner (each 770 × 600 µm, at 20×) is larger than that of a Vectra scanner. *** The analysis of fewer regions of interest is possible but warrants cautious interpretation of the data. ^†^ Each sample needs to be evaluated individually.

## Supplementary Materials

The following are available online at https://www.mdpi.com/2072-6694/12/2/255/s1, Figure S1: Tyramide signal amplification workflow, Figure S2: Western blot and microphotographs of representative examples from PD-L1 clones validation, Figure S3: Spectral library creation with the different fluorophos from the Opal-7 kit, Figure S4: Storage alterations, Figure S5: Tissue segmentation workflow using InForm image analysis software, Figure S6: Cell segmentation workflow using InForm image analysis software, Figure S7: Phenotyping workflow strategy using InForm image analysis software to identify each marker allocate in a multiplex panel, Figure S8: Spatial distribution analysis, Figure S9: Multiplex immunofluorescence (mIF) microphotographs using different multiplex panel for immune profiling several types of tumor histologies, Table S1: Immune profiling cases using multiplex immunofluorescence from 2015 to 2018 at the Translational Molecular Pathology Immunoprofiling Laboratory.

## References

[1] Devaud C., John L.B., Westwood J.A., Darcy P.K., Kershaw M.H. (2013). Immune modulation of the tumor microenvironment for enhancing cancer immunotherapy. Oncoimmunology.

[2] Tang H., Qiao J., Fu Y.X. (2016). Immunotherapy and tumor microenvironment. Cancer Lett..

[3] Dougan M., Dougan S.K. (2017). Targeting Immunotherapy to the Tumor Microenvironment. J. Cell Biochem..

[4] Frankel T., Lanfranca M.P., Zou W. (2017). The Role of Tumor Microenvironment in Cancer Immunotherapy. Adv. Exp. Med. Biol..

[5] Gajewski T.F., Corrales L., Williams J., Horton B., Sivan A., Spranger S. (2017). Cancer Immunotherapy Targets Based on Understanding the T Cell-Inflamed Versus Non-T Cell-Inflamed Tumor Microenvironment. Adv. Exp. Med. Biol..

[6] Cauwels A., Van Lint S., Garcin G., Bultinck J., Paul F., Gerlo S., Van der Heyden J., Bordat Y., Catteeuw D., De Cauwer L. (2018). A safe and highly efficient tumor-targeted type I interferon immunotherapy depends on the tumor microenvironment. Oncoimmunology.

[7] Dixon A.R., Bathany C., Tsuei M., White J., Barald K.F., Takayama S. (2015). Recent developments in multiplexing techniques for immunohistochemistry. Expert Rev. Mol. Diagn..

[8] Blom S., Paavolainen L., Bychkov D., Turkki R., Maki-Teeri P., Hemmes A., Valimaki K., Lundin J., Kallioniemi O., Pellinen T. (2017). Systems pathology by multiplexed immunohistochemistry and whole-slide digital image analysis. Sci. Rep..

[9] Surace M., DaCosta K., Huntley A., Zhao W., Bagnall C., Brown C., Wang C., Roman K., Cann J., Lewis A. (2019). Automated Multiplex Immunofluorescence Panel for Immuno-oncology Studies on Formalin-fixed Carcinoma Tissue Specimens. J. Vis. Exp..

[10] Parra E.R. (2018). Novel Platforms of Multiplexed Immunofluorescence for Study of Paraffin Tumor Tissues. J. Cancer Treat. Diagn..

[11] Steiner C., Ducret A., Tille J.C., Thomas M., McKee T.A., Rubbia-Brandt L., Scherl A., Lescuyer P., Cutler P. (2014). Applications of mass spectrometry for quantitative protein analysis in formalin-fixed paraffin-embedded tissues. Proteomics.

[12] Stauber J., MacAleese L., Franck J., Claude E., Snel M., Kaletas B.K., Wiel I.M., Wisztorski M., Fournier I., Heeren R.M. (2010). On-tissue protein identification and imaging by MALDI-ion mobility mass spectrometry. J. Am. Soc. Mass Spectrom..

[13] Sood A., Miller A.M., Brogi E., Sui Y., Armenia J., McDonough E., Santamaria-Pang A., Carlin S., Stamper A., Campos C. (2016). Multiplexed immunofluorescence delineates proteomic cancer cell states associated with metabolism. JCI Insight.

[14] Gorris M.A.J., Halilovic A., Rabold K., van Duffelen A., Wickramasinghe I.N., Verweij D., Wortel I.M.N., Textor J.C., de Vries I.J.M., Figdor C.G. (2018). Eight-Color Multiplex Immunohistochemistry for Simultaneous Detection of Multiple Immune Checkpoint Molecules within the Tumor Microenvironment. J. Immunol..

[15] Rost S., Giltnane J., Bordeaux J.M., Hitzman C., Koeppen H., Liu S.D. (2017). Multiplexed ion beam imaging analysis for quantitation of protein expresssion in cancer tissue sections. Lab. Investig..

[16] Hofman P., Badoual C., Henderson F., Berland L., Hamila M., Long-Mira E., Lassalle S., Roussel H., Hofman V., Tartour E. (2019). Multiplexed Immunohistochemistry for Molecular and Immune Profiling in Lung Cancer-Just About Ready for Prime-Time?. Cancers.

[17] Shipitsin M., Small C., Giladi E., Siddiqui S., Choudhury S., Hussain S., Huang Y.E., Chang H., Rimm D.L., Berman D.M. (2014). Automated quantitative multiplex immunofluorescence in situ imaging identifies phospho-S6 and phospho-PRAS40 as predictive protein biomarkers for prostate cancer lethality. Proteome Sci..

[18] Xie R., Chung J.Y., Ylaya K., Williams R.L., Guerrero N., Nakatsuka N., Badie C., Hewitt S.M. (2011). Factors influencing the degradation of archival formalin-fixed paraffin-embedded tissue sections. J. Histochem. Cytochem..

[19] Bobrow M.N., Harris T.D., Shaughnessy K.J., Litt G.J. (1989). Catalyzed reporter deposition, a novel method of signal amplification. Application to immunoassays. J. Immunol. Methods.

[20] Bobrow M.N., Shaughnessy K.J., Litt G.J. (1991). Catalyzed reporter deposition, a novel method of signal amplification. II. Application to membrane immunoassays. J. Immunol. Methods.

[21] Parra E.R., Uraoka N., Jiang M., Cook P., Gibbons D., Forget M.A., Bernatchez C., Haymaker C., Wistuba I.I., Rodriguez-Canales J. (2017). Validation of multiplex immunofluorescence panels using multispectral microscopy for immune-profiling of formalin-fixed and paraffin-embedded human tumor tissues. Sci. Rep..

[22] Lin F., Prichard J. (2015). Handbook of Practical Immunohistochemistry: Frequently Asked Questions.

[23] Parra E.R., Villalobos P., Mino B., Rodriguez-Canales J. (2018). Comparison of Different Antibody Clones for Immunohistochemistry Detection of Programmed Cell Death Ligand 1 (PD-L1) on Non-Small Cell Lung Carcinoma. Appl. Immunohistochem. Mol. Morphol..

[24] Acharya P., Quinlan A., Neumeister V. (2017). The ABCs of finding a good antibody: How to find a good antibody, validate it, and publish meaningful data. F1000Research.

[25] O’Hurley G., Sjostedt E., Rahman A., Li B., Kampf C., Ponten F., Gallagher W.M., Lindskog C. (2014). Garbage in, garbage out: A critical evaluation of strategies used for validation of immunohistochemical biomarkers. Mol. Oncol..

[26] Wolff A.C., Hammond M.E.H., Allison K.H., Harvey B.E., Mangu P.B., Bartlett J.M.S., Bilous M., Ellis I.O., Fitzgibbons P., Hanna W. (2018). Human Epidermal Growth Factor Receptor 2 Testing in Breast Cancer: American Society of Clinical Oncology/College of American Pathologists Clinical Practice Guideline Focused Update. Arch. Pathol. Lab. Med..

[27] Hammond M., Hayes D., Dowsett M. (2010). Pathologists’ Guideline Recommendations for Immunohistochemical Testing of Estrogen and Progesterone Receptors in Breast Cancer. Breast Care.

[28] Bordeaux J., Welsh A., Agarwal S., Killiam E., Baquero M., Hanna J., Anagnostou V., Rimm D. (2010). Antibody validation. Biotechniques.

[29] Parra E.R., Villalobos P., Rodriguez-Canales J. (2019). The Multiple Faces of Programmed Cell Death Ligand 1 Expression in Malignant and Nonmalignant Cells. Appl. Immunohistochem. Mol. Morphol..

[30] Carvajal-Hausdorf D.E., Schalper K.A., Neumeister V.M., Rimm D.L. (2015). Quantitative measurement of cancer tissue biomarkers in the lab and in the clinic. Lab. Investig..

[31] Fitzgibbons P.L., Bradley L.A., Fatheree L.A., Alsabeh R., Fulton R.S., Goldsmith J.D., Haas T.S., Karabakhtsian R.G., Loykasek P.A., Marolt M.J. (2014). Principles of analytic validation of immunohistochemical assays: Guideline from the College of American Pathologists Pathology and Laboratory Quality Center. Arch. Pathol. Lab. Med..

[32] Parra E.R., Francisco-Cruz A., Wistuba I.I. (2019). State-of-the-Art of Profiling Immune Contexture in the Era of Multiplexed Staining and Digital Analysis to Study Paraffin Tumor Tissues. Cancers.

[33] Francisco-Cruz A., Parra E.R., Tetzlaff M.T., Wistuba I.I. (2020). Multiplex Immunofluorescence Assays. Methods Mol. Biol..

[34] Gown A.M. (2004). Unmasking the mysteries of antigen or epitope retrieval and formalin fixation. Am. J. Clin. Pathol..

[35] Machaalani R., Radford J.L., Waters K.A. (2007). Tissue fixation effects on immunohistochemical staining of caspase-3 in brain tissue. Appl. Immunohistochem. Mol. Morphol..

[36] Yildiz-Aktas I.Z., Dabbs D.J., Bhargava R. (2012). The effect of cold ischemic time on the immunohistochemical evaluation of estrogen receptor, progesterone receptor, and HER2 expression in invasive breast carcinoma. Mod. Pathol..

[37] Apple S., Pucci R., Lowe A.C., Shintaku I., Shapourifar-Tehrani S., Moatamed N. (2011). The effect of delay in fixation, different fixatives, and duration of fixation in estrogen and progesterone receptor results in breast carcinoma. Am. J. Clin. Pathol..

[38] Hammond M.E., Hayes D.F., Wolff A.C., Mangu P.B., Temin S. (2010). American society of clinical oncology/college of american pathologists guideline recommendations for immunohistochemical testing of estrogen and progesterone receptors in breast cancer. J. Oncol. Pract..

[39] Parra E.R., Streckfus C.F. (2018). Immune Cell Profiling in Cancer Using Multiplex Immunofluorescence and Digital Analysis Approaches.

[40] Aeffner F., Zarella M.D., Buchbinder N., Bui M.M., Goodman M.R., Hartman D.J., Lujan G.M., Molani M.A., Parwani A.V., Lillard K. (2019). Introduction to Digital Image Analysis in Whole-slide Imaging: A White Paper from the Digital Pathology Association. J. Pathol. Inform..

[41] Pell R., Oien K., Robinson M., Pitman H., Rajpoot N., Rittscher J., Snead D., Verrill C., UK National Cancer Research Institute (NCRI) Cellular-Molecular Pathology (CM-Path) Quality Assurance Working Group (2019). The use of digital pathology and image analysis in clinical trials. J. Pathol. Clin. Res..

[42] Stack E.C., Wang C., Roman K.A., Hoyt C.C. (2014). Multiplexed immunohistochemistry, imaging, and quantitation: A review, with an assessment of Tyramide signal amplification, multispectral imaging and multiplex analysis. Methods.

[43] Werner M., Chott A., Fabiano A., Battifora H. (2000). Effect of formalin tissue fixation and processing on immunohistochemistry. Am. J. Surg. Pathol..

[44] Mirlacher M., Kasper M., Storz M., Knecht Y., Durmuller U., Simon R., Mihatsch M.J., Sauter G. (2004). Influence of slide aging on results of translational research studies using immunohistochemistry. Mod. Pathol..

[45] Grillo F., Bruzzone M., Pigozzi S., Prosapio S., Migliora P., Fiocca R., Mastracci L. (2017). Immunohistochemistry on old archival paraffin blocks: Is there an expiry date?. J. Clin. Pathol..

[46] Hewitt S.M., Lewis F.A., Cao Y., Conrad R.C., Cronin M., Danenberg K.D., Goralski T.J., Langmore J.P., Raja R.G., Williams P.M. (2008). Tissue handling and specimen preparation in surgical pathology: Issues concerning the recovery of nucleic acids from formalin-fixed, paraffin-embedded tissue. Arch. Pathol. Lab. Med..

[47] Parra E.R., Villalobos P., Behrens C., Jiang M., Pataer A., Swisher S.G., William W.N., Zhang J., Lee J., Cascone T. (2018). Effect of neoadjuvant chemotherapy on the immune microenvironment in non-small cell lung carcinomas as determined by multiplex immunofluorescence and image analysis approaches. J. Immunother. Cancer.

[48] Barua S., Solis L., Parra E.R., Uraoka N., Jiang M., Wang H., Rodriguez-Canales J., Wistuba I., Maitra A., Sen S. (2018). A Functional Spatial Analysis Platform for Discovery of Immunological Interactions Predictive of Low-Grade to High-Grade Transition of Pancreatic Intraductal Papillary Mucinous Neoplasms. Cancer Inform..

[49] Huang W., Hennrick K., Drew S. (2013). A colorful future of quantitative pathology: Validation of Vectra technology using chromogenic multiplexed immunohistochemistry and prostate tissue microarrays. Hum. Pathol..

[50] Nederlof M., Watanabe S., Burnip B., Taylor D.L., Critchley-Thorne R. (2011). High-throughput profiling of tissue and tissue model microarrays: Combined transmitted light and 3-color fluorescence digital pathology. J. Pathol. Inform..

[51] Hoyt C., Roman K., Engle L., Wang C., Ballesteros-Merino C., Jensen S., McGuire J., Zheng Y., Coltharp C., Jiang M. (2019). Abstract LB-318: Multi-institutional TSA-amplified Multiplexed Immunofluorescence Reproducibility Evaluation (MITRE study): Reproducibility assessment of an automated multiplexed immunofluorescence slide staining, imaging, and analysis workflow. Cancer Res..

[52] Roy S., Axelrod H.D., Valkenburg K.C., Amed S., Pienta K.J. (2019). Optimization of prostate cancer cell detection using multiplex tyramide signal amplification. J. Cell Biochem..

