# Supplementary material for: Procedural Requirements and Recommendations for Multiplex Immunofluorescence Tyramide Signal Amplification Assays to Support Translational Oncology Studies
Source: Cancers (Basel). 2020 Jan 21;12(2):255. doi: 10.3390/cancers12020255 (PMC7072187; doi:10.3390/cancers12020255)
Supplement: Supplementary file 1 [file cancers-12-00255-s001.pdf]

Supplementary Figure 1

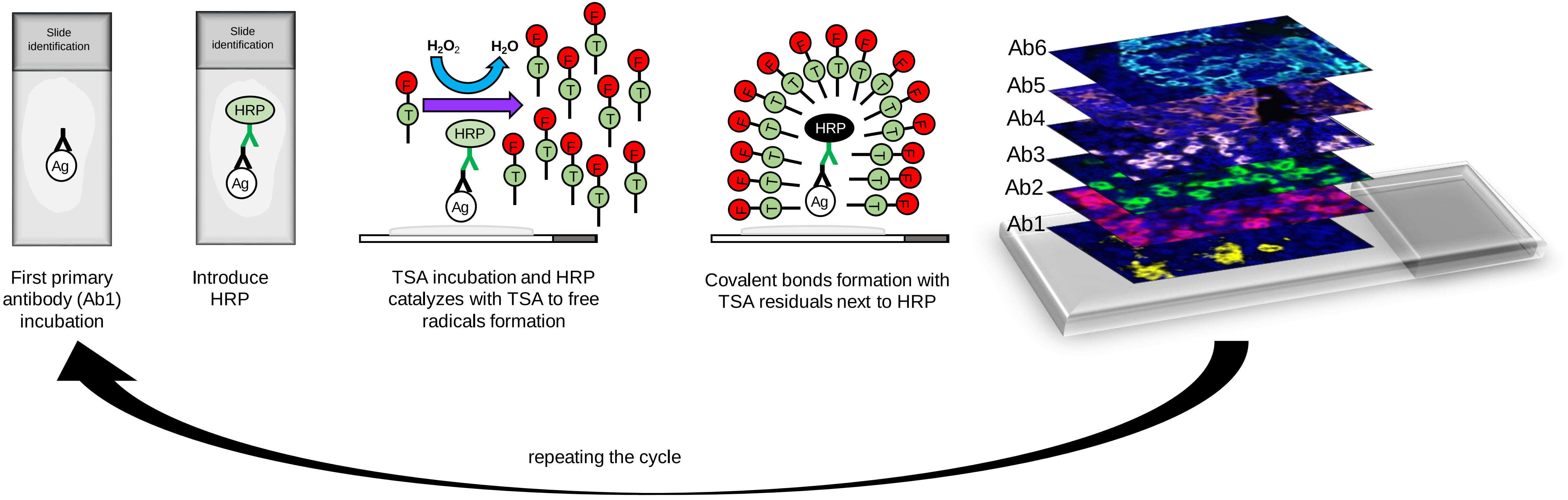

Supplementary Figure 2

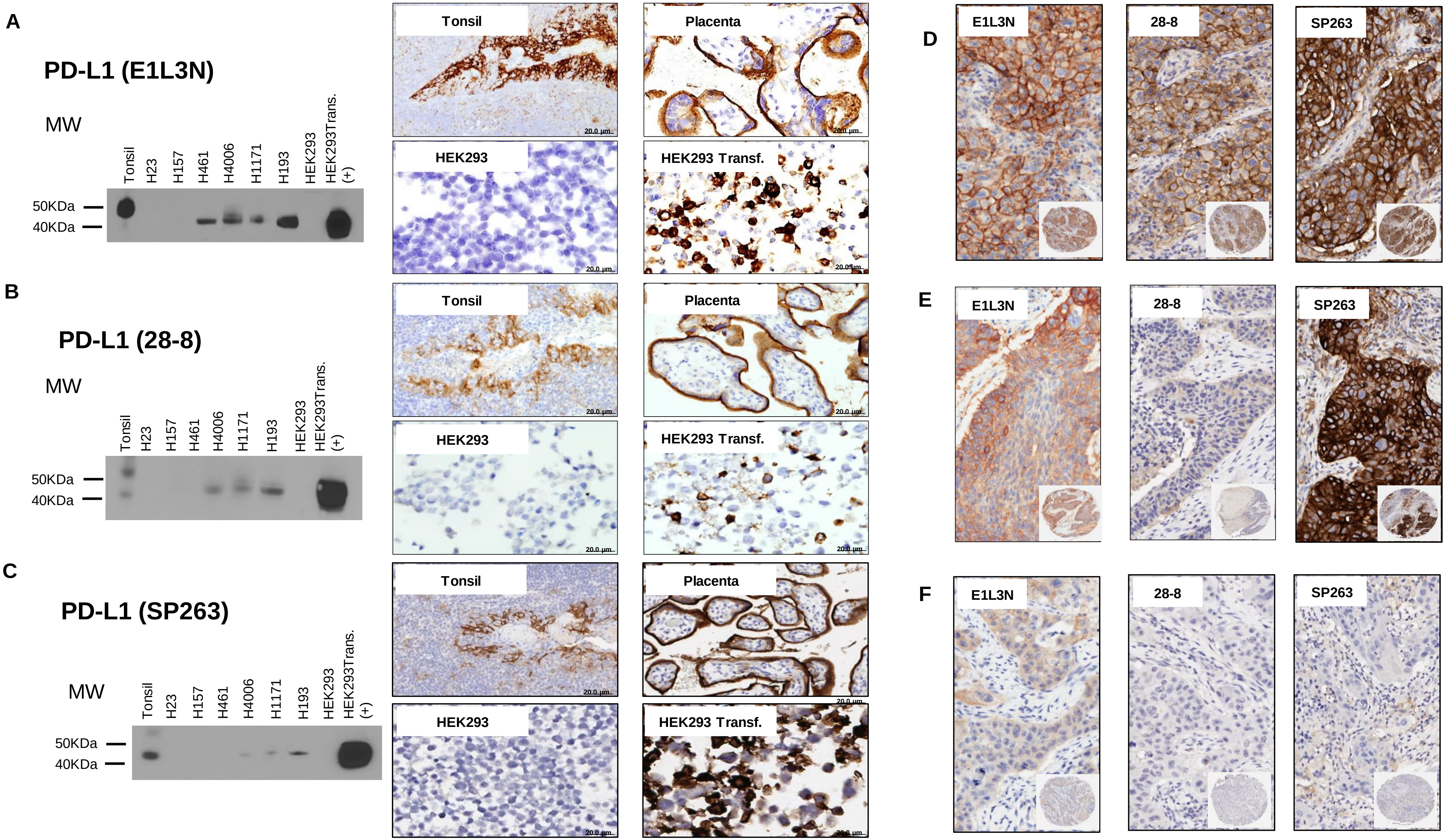

Supplementary Figure 3

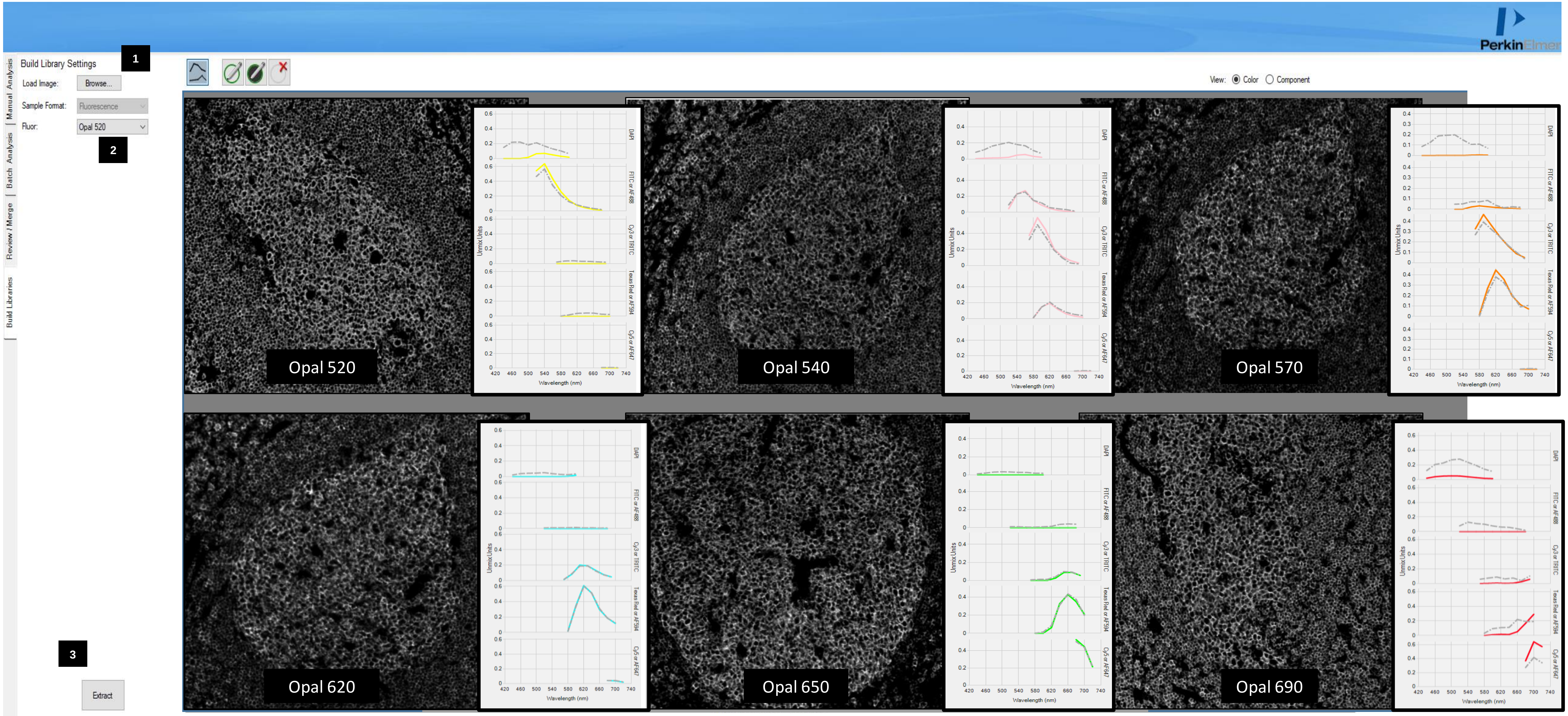

Supplementary Figure 4

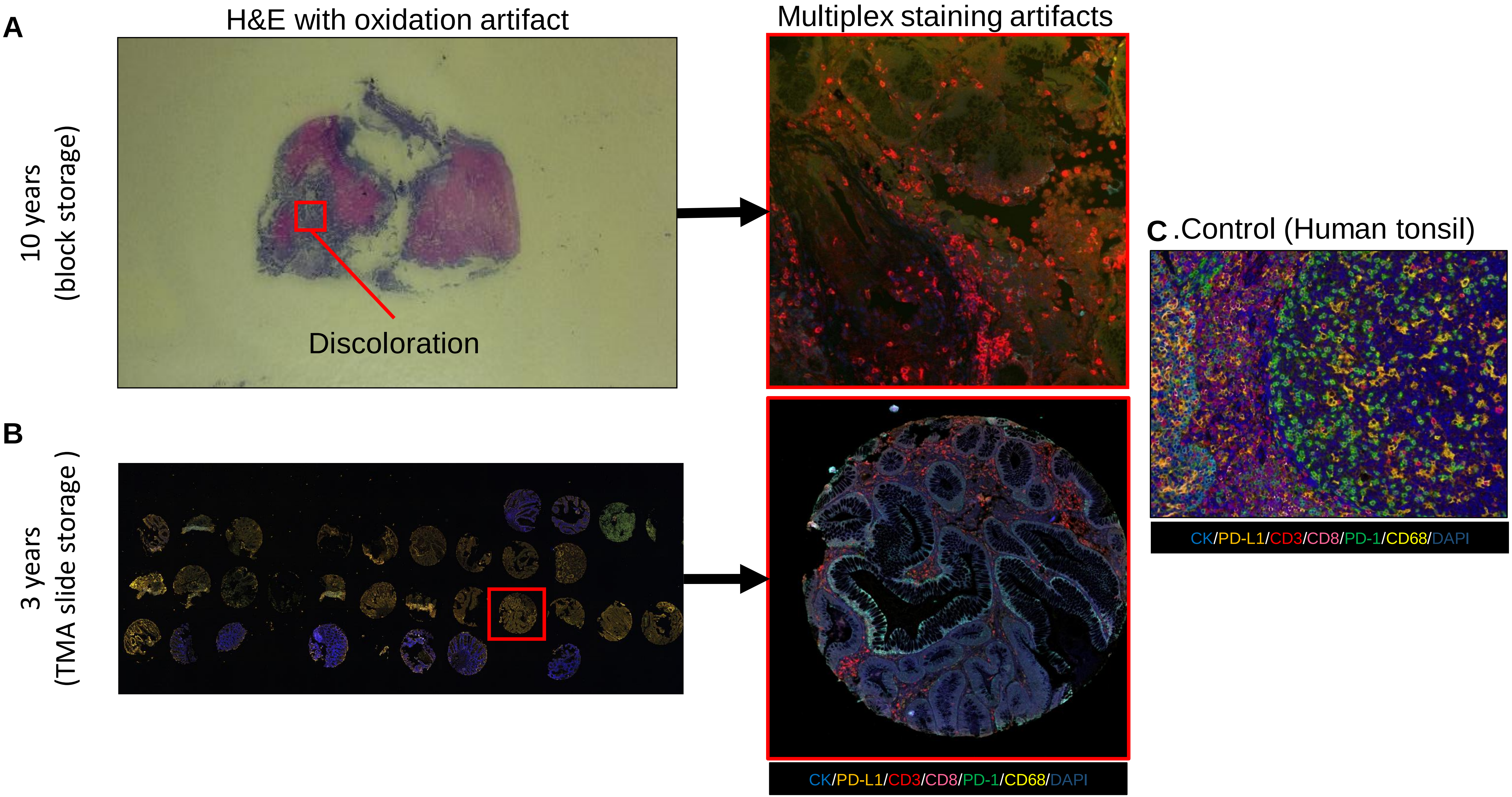

Supplementary Figure 5

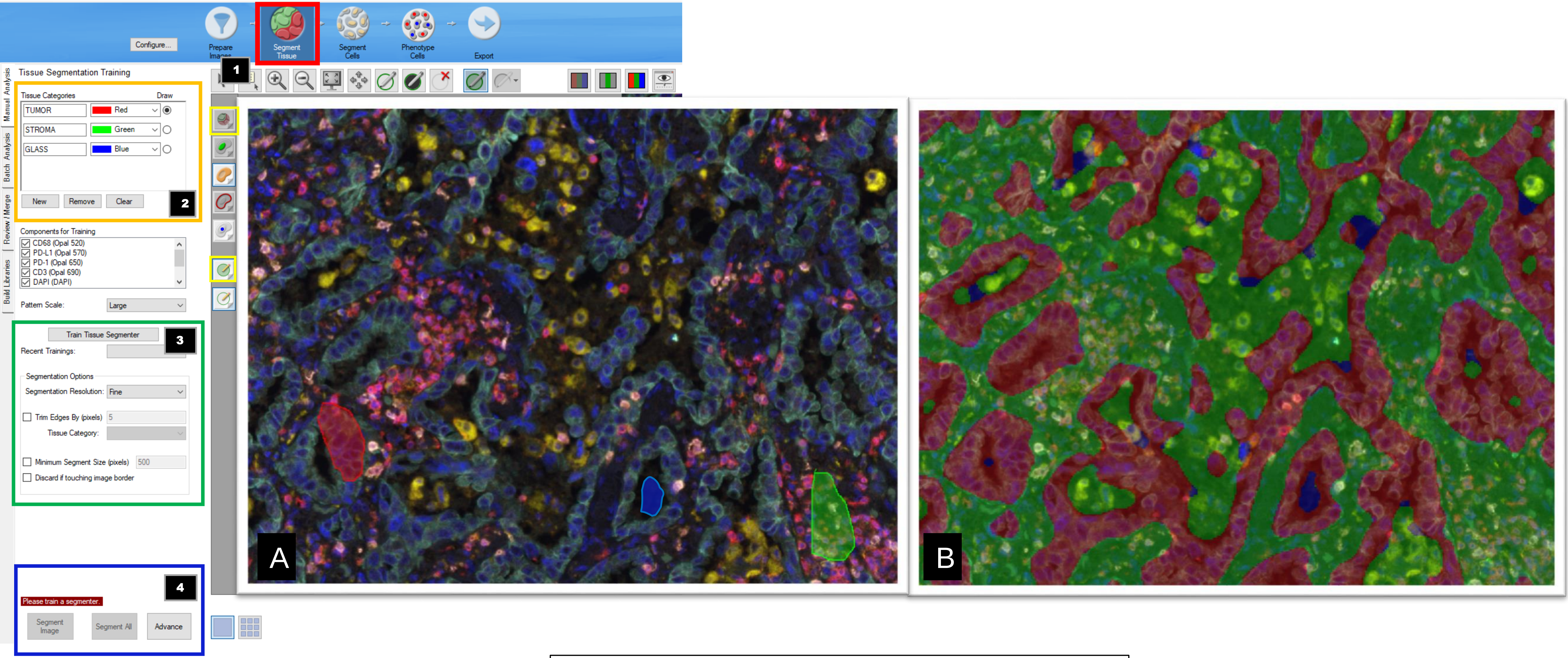

Tissue segmentation visualization tools

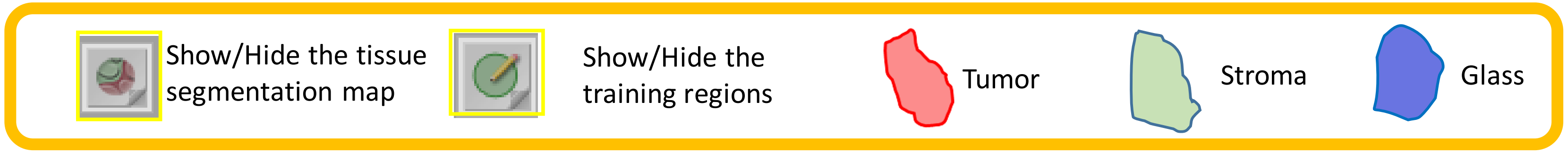

Supplementary Figure 6

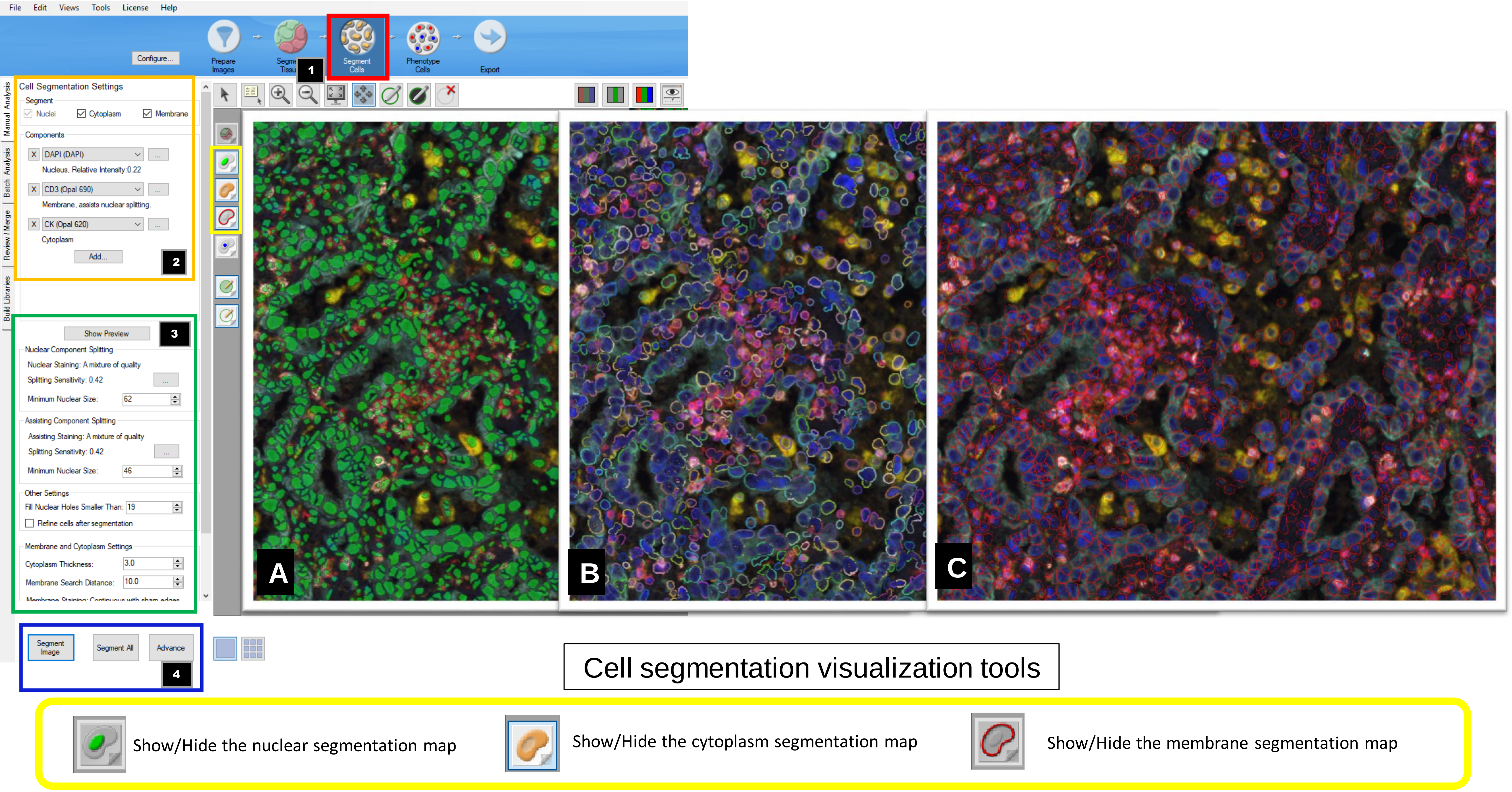

Supplementary Figure 7

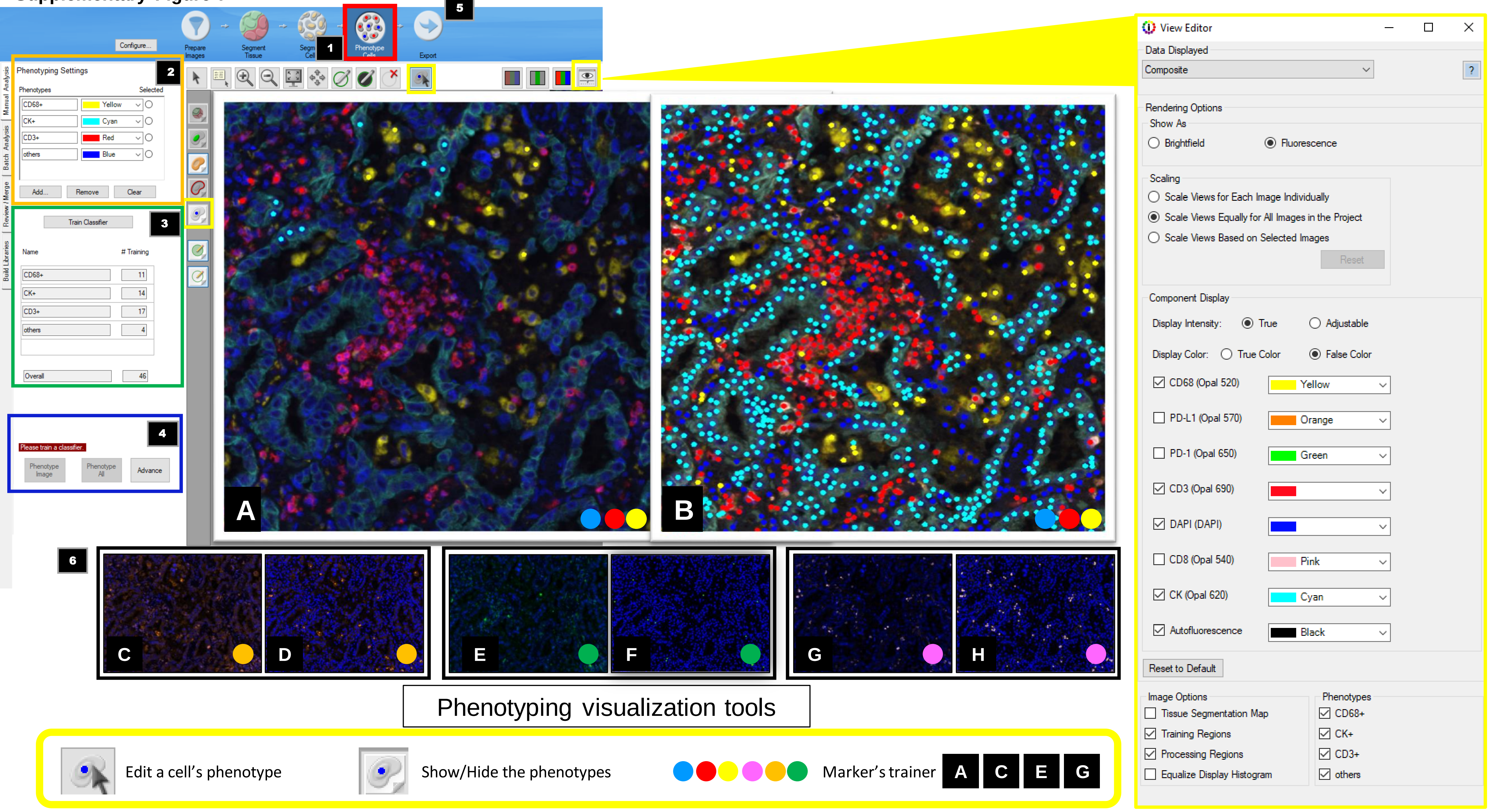

Supplementary Figure 8

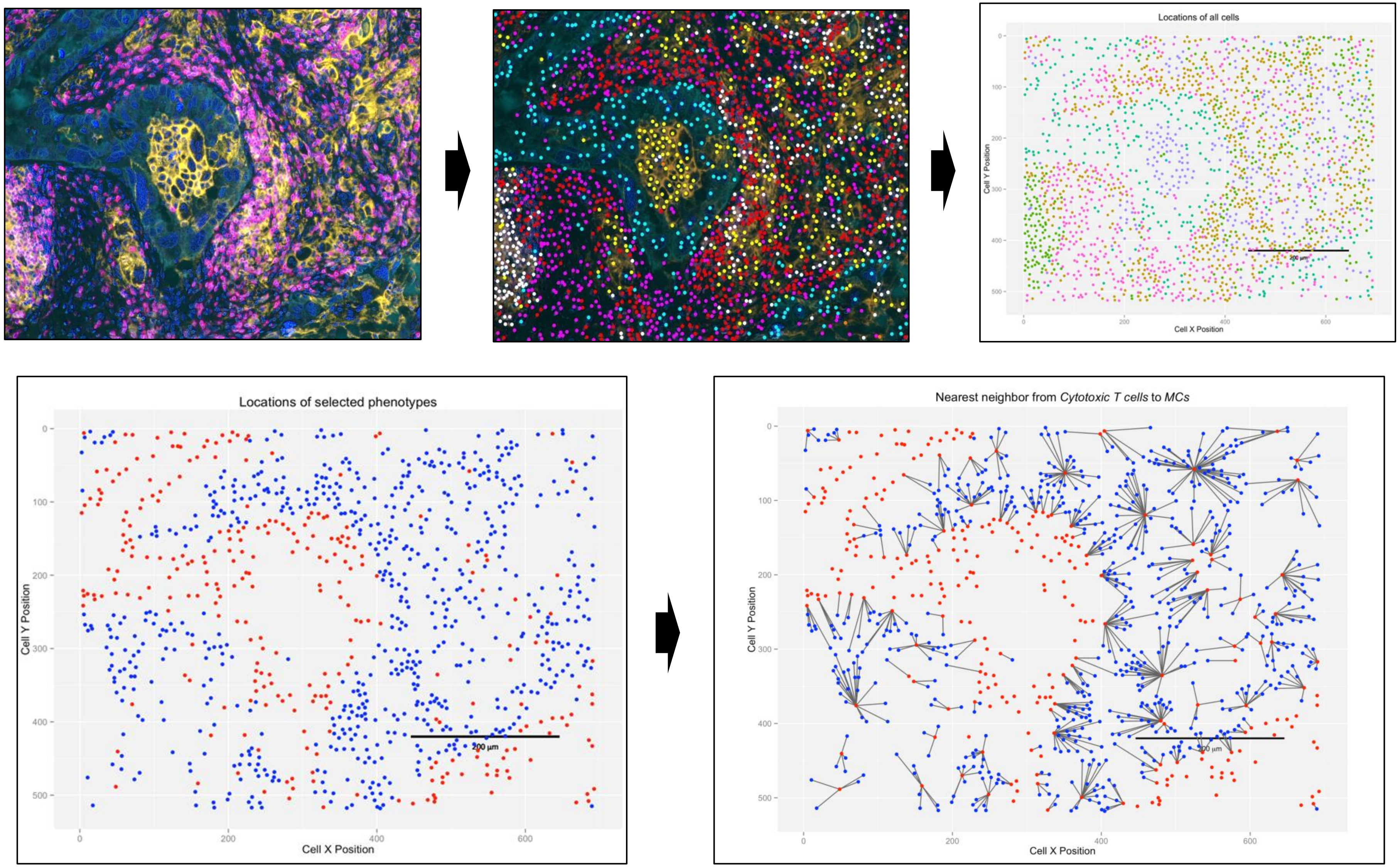

CD3+/CD8+ Cytotoxic T Cells

Malignant Cells

Mean Distance Between Cytotoxic T cells to MCs = 285.9 microns

Supplementary Figure 9

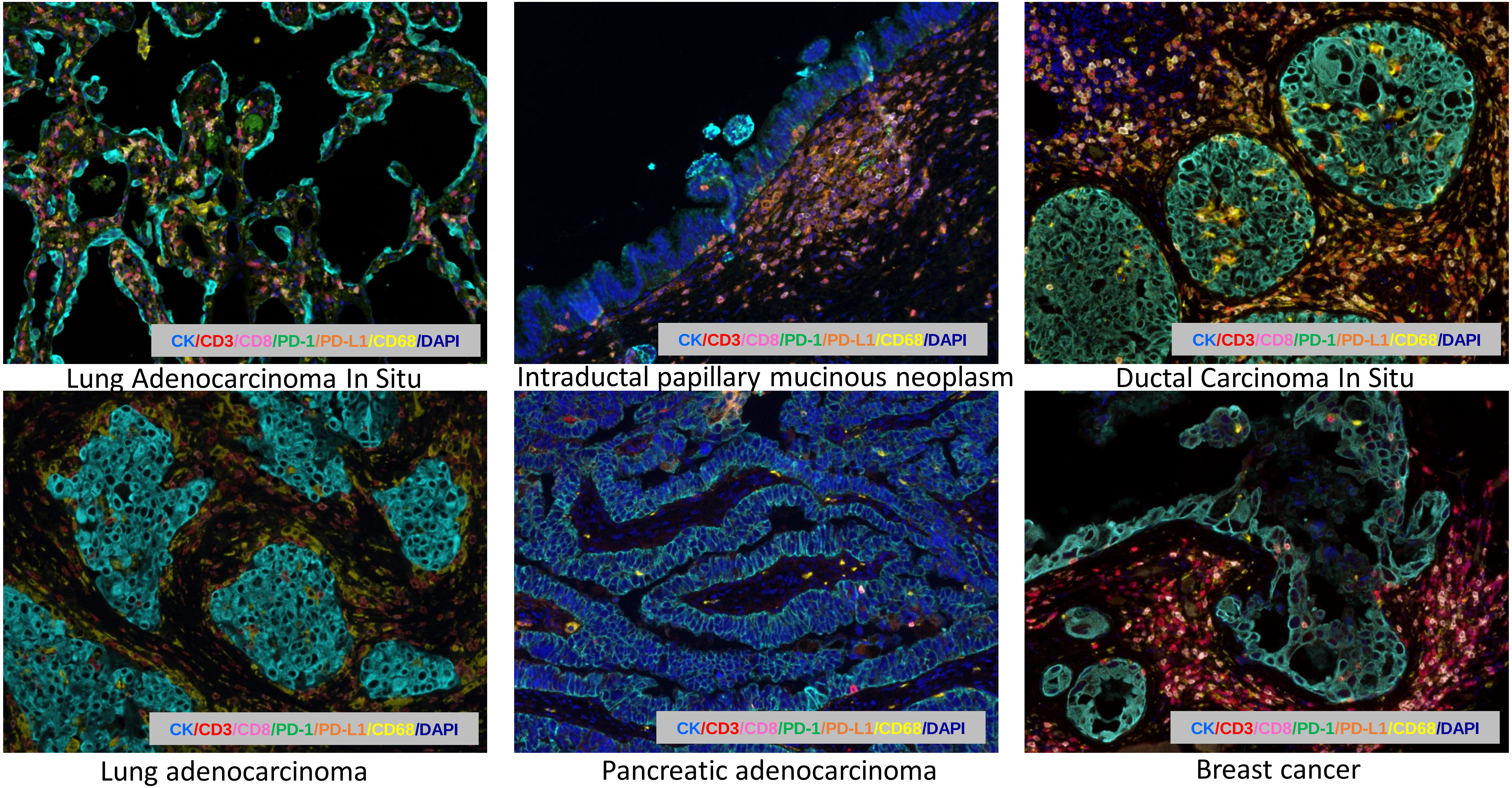

Supplementary Table 1. Immune profiling cases using multiplex immunofluorescence from 2015 to 2018 in our Immunoprofiling Laboratory.

Organ	Histology type	Cases (N)	Slides (N)	Tissue category	Panel (N)	ROIs (N)
Lung	ADC/SCC	829	1503	WS/CNB/TMA	5	7774
Mesothelium	Mesothelioma	52	104	CNB	2	520
Head and neck	SCC	269	332	WS	4	1660
Esophagus	SCC	22	44	WS	2	220
Liver	HCC	15	30	WS	2	150
Colon/rectum	CRC	172	313	CNB/TMA	3	1565
Ovary	ADC	240	10	TMA	2	240
Breast	ADC/DCIS	144	249	WS	2	1245
Soft tissue	Sarcoma	278	556	CNB	2	2780
Brain	GBM	43	82	WS	2	410
Skin	Merkel cell carcinoma	82	82	WS	1	410
Pancreas	IPMN	130	260	WS	2	1300
Other	Miscellaneous	238	577	WS/CNB	2	2885
**Total**	**11**	**2514**	**4142**	**3**	**5**	**21159**

ADC, adenocarcinoma; SCC, squamous cell carcinoma; HCC, hepatocellular carcinoma; CRC, colorectal carcinoma; DCIS, ductal carcinoma in situ; GBM, glioblastoma; IPMN, intraductal papillary mucinous neoplasm of the pancreas; WS, whole section; CNB, core-needle biopsy; TMA, tissue microarray; ROI, region of interest.
